# Association of Marshall CT Scores with GFAP, UCH-L1, Tau, NfL, and p-Tau231 After Traumatic Brain Injury

**DOI:** 10.3390/ijms262411765

**Published:** 2025-12-05

**Authors:** Katie A. Edwards, John Alice, Maryn Day, Joseph Yun, Sijung Yun, Heather E. Dark, Lillian Gabor, Jessica M. Gill

**Affiliations:** 1 School of Nursing, Johns Hopkins University, Baltimore, MD 21218, USAsyun16@jhu.edu (S.Y.);; 2 School of Medicine, Neurology, Johns Hopkins University, Baltimore, MD 21218, USA

**Keywords:** biomarkers, traumatic brain injury, Marshall CT classification, Glasgow Coma Scale, glial fibrillary acid protein, neurofilament light chain, phosphorylated tau

## Abstract

This study identifies a significant association among blood-based biomarkers of traumatic brain injury (TBI) and the Marshall CT classification of TBI (MCTC) scores, but not with Glasgow Coma Scale (GCS) scores. We aimed to determine whether GCS and MCTC scores relate to glial fibrillary acid protein (GFAP), ubiquitin carboxy hydrolase-1 (UCH-L1), tau, neurofilament light chain (NfL), and phosphorylated tau (p-tau231) concentrations following acute TBIs. Participants included patients from 20 trauma centers across 12 regional sites in the United States and Canada with an initial CT scan within 6 h after TBI and GCS scores of 3 to 12. Blood samples collected upon hospital arrival were analyzed for biomarker concentrations (pg/mL). Concentrations from 271 patients with GCS ≥ 9 were compared to 145 with GCS ≤ 9. Samples from 347 patients with MCTC < 3 were compared to 70 with MCTC ≥ 3. No significant differences in GCS groups were found (*p*’s > 0.5), while MCTC groups differed significantly (*p*’s < 0.001). Higher concentrations of plasma GFAP, NfL, and p-tau231 correlated with MCTC scores > 3, with no associations with GCS. Future research might show an application in individual risk assessments to improve triaging of TBI patients.

## 1. Introduction

Over 2 million Americans require immediate care for traumatic brain injuries (TBIs) each year in the United States, with morbidity and mortality rates that remain critically high [1,2]. Current diagnostic tools to guide clinical decisions in the acute care setting rely highly on computer tomography (CT) imaging, with blood-based biomarkers having limitations related to sensitivity and specificity that are not optimal, introducing significant challenges at important timepoints along the patient’s care pathway [3]. The Federal Drug Administration (FDA) approved glial fibrillary acid protein (GFAP) and ubiquitin carboxyl termina hydrolase-1 (UCH-L1) in 2018 to determine the need for CT utilization in patients with suspected TBI, thus providing an initial platform to improve diagnostic decision-making, particularly in emergency care settings. Since this approval, a number of large observational studies have supported this finding in patients with a mild Glasgow Coma Score (GCS) of 13–15, as well as expanding application in TBI patients across the severity spectrum [4,5,6]. Further, higher levels of UCH-L1, GFAP, tau, and neurofilament light chain (NfL) have been associated with sustaining a TBI and consistently associated with TBIs across severities [7,8,9,10,11,12,13]. These studies provide the foundation to expand the context of uses for fluid biomarkers for GFAP and UCLH1, as well as in identifying additional biomarkers able to improve sensitivity and specificity within TBI clinical cohorts.

Specifically, GFAP and UCHL-1 in addition to NfL and tau proteins are candidate biomarkers that have been well-characterized in TBI. GFAP, an astrocyte-enriched intermediate filament, and UCH-L1, a neuron-specific deubiquitinating enzyme, increase rapidly in the hours after injury and on the day of injury show good-to-excellent performance for detecting intracranial lesions and predicting 6-month unfavorable outcomes and mortality [7,9,14,15,16]. NfL, a structural protein found in myelinated axons, rises within the first day after injury and continues to increase over the following days to weeks, reflecting axonal damage and showing associations with functional recovery and long-term outcomes [13,15,16,17]. Tau proteins, which stabilize neuronal microtubules, and phosphorylated tau species (e.g., p-tau231, an emerging TBI biomarker) reflect neuronal cytoskeletal disruption and hyperphosphorylation, contributing to neurodegeneration and cognitive decline [7]. Recent work indicates that serum brain-derived tau, serum total tau, and p-tau231 peak within the first hours to two days following injury, and show differential associations with clinical outcomes over one year after severe TBI, suggesting potential complementary utility alongside astroglial and axonal markers [7,18,19]. Taken together, increases in GFAP/UCH-L1 concentrations within hours primarily index acute astroglial and neuronal injury, while subsequent increases in tau/p-tau species and NfL track evolving microtubule and axonal pathology, respectively—patterns that align with increasing injury severity and worse prognosis across cohorts.

Severe traumatic brain injury (sTBI) in particular remains a leading cause of death and long-term disability worldwide and there is an urgent need for reliable, minimally invasive biomarkers to improve early diagnosis, guide clinical decision-making, and ultimately improve long-term outcomes [20]. The GCS is an accessible clinical measure for assessing TBI severity. The GCS was first published in 1974 as a standardized method for evaluating consciousness following TBI [21], and it has since become considered the gold standard for documenting and communicating overall injury severity. The GCS total score is stratified into mild (GCS = 13–15), moderate (GCS = 9–12), or severe (GCS ≤ 8) classification based on eye, verbal, and motor responses. While studies have demonstrated that lower GCS scores (GCS ≤ 8) are associated with worse outcomes following TBI [22,23,24], the GCS carries well-recognized limitations as an indicator of structural/cellular brain damage and outcomes because it is influenced by factors such as intoxication, sedation, intubation, polytrauma (i.e., extracranial injury), communication (i.e., language barrier, intellectual or neurological deficit, hearing loss or speech impediment), and delayed assessment [24,25,26,27,28]. Thus, incorporating complementary severity scoring systems and clinical assessments, which may include neuroimaging and fluid biomarkers, is crucial for accurately assessing patient outcomes [22,27,29].

Although severe TBIs are far easier to diagnose than injuries with fewer pathological processes and clinical presentations that are more clear, aligning fluid biomarkers with imaging outcomes that have clinical significance would lead to opportunities to integrate fluid biomarkers into clinical decisions, possibly leading to improved outcomes that have been observed in stroke diagnosis and clinical management. The Marshall CT classification of traumatic brain injury may be a method to develop this opportunity. Originally published in 1992, the MCTC is a CT scan-based score commonly used in TBI assessment (Table A1). The assessment considers features such as presence of mass lesions, signs of raised intracranial pressure (e.g., mesencephalic cistern compression, midline shift), and surgical evacuation of a lesion to determine TBI severity [30]. Contrary to a CT+/CT− classification of CT scans, the MCTC is an ordinal scale with unequal intervals with a range from 0 to 6, where TBI severity increases as the score increases; an MCTC score below 4 has been previously associated with improved survival outcomes in patients and fewer deaths are reported in patients with a score of 5 when compared to 6 [31,32,33]. Dichotomization at ≥3 vs. <3 distinguishes diffuse injury (Classes I–II) from structural injury with mass effect or compressed cisterns (Classes III–VI), which carry higher mortality and unfavorable outcomes, and has been frequently used in biomarker and prognostic studies [32,34,35,36,37,38,39,40,41]. While the MCTC provides a more nuanced understanding of CT scan findings compared to a CT+/CT− classification, it is not feasible to order a CT scan or repeated scans in every setting. This limitation necessitates the identification of a more accessible tool that can provide an approximation of this metric.

In this manuscript, we elected to utilize the Marshall CT scoring method. Although CT imaging has certain limitations, emerging approaches that estimate the risk of mortality, morbidity, and evolving secondary pathology offer valuable pathways for improving fluid biomarker panels used in diagnosis and prognosis. One of these methods is the Marshall CT score. In a study of over 1000 severe TBI patients, area under the curve (AUC) models indicated that among the most common CT scoring systems, the MCTC was somewhat better in predicting early unfavorable outcomes (Marshall AUC 0.86 vs. Rotterdam AUC 0.82 vs. Helsinki AUC 0.84). Further, a high correlation was found between Marshall and Rotterdam grading, r = 0.78, and to have a moderate correlation between the other two pairs (Marshall vs. Helsinki, r = 0.62, and Rotterdam vs. Helsinki, r = 0.51) [42]. In a similar study, the MCTC and neurological radiological interpretation system (NIRIS) scoring systems had higher AUCs for predicting ICU admission and required neurosurgery than the other scoring systems, as well as the most common indicators of recovery, the Glasgow Outcomes Score Extended (GOSE) at 6 months [43]. Further, the MCTC is highly associated with acute mortality, as well as in imaging evidence of specific sub-types that required interventions. In moderate and severe TBIs, the mortality in patients with a score of 1 and 2 is 0%, a score of 3 is 90%, a score of 4 is 31.97%, a score of 5 is 31.97%, and a score of 6 is 100% [34].

Current studies in TBI biomarkers have primarily focused on differentiating CT+ and CT− TBI patients, neglecting the clinical reality that CT findings are not used in a clear dichotomous manner, and instead used to determine the severity of injuries, as well as for monitoring pathological changes, and most importantly determining the need for surgical interventions [44]. Specifically, CT imaging provides evidence of injury sub-types, which include the following: skull fracture (SF), subdural hematoma (SDH), intraparenchymal hemorrhage (IH), epidermal hematoma (EH), micro-bleeds (MDs), cerebral contusions (CC), and cerebral edema (CE), as well as the location of these injuries and severities. Further complicating the current paradigm of CT-based biomarkers is that CT findings are often plural in nature, in that few patients have evidence of only one CT imaging sub-type, and thus more qualitive and integrative interpretation should be applied to the development of fluid biomarkers to extend their potential benefit by aligning with clinical interpretations of risks. Thus, acute care is focused on preserving life and mitigating secondary injury progression, leading to further compromises in neurological functioning, and minimizing biomarker identification to focus primarily on distinguishing CT+ vs. CT− has limited the progression of biomarkers to inform acute care decisions. We suggest that aligning biomarkers with the categorization of severities and sub-types based on imaging coding such as the Marshall CT scoring will progress the development of fluid biomarkers that can influence practice.

## 2. Results

Demographic and clinical characteristics are presented in Table 1 and Table 2. A total of 271 patients with GCS ≥ 9 (207 male [74.5%]; mean [SD] age at baseline: 44.4 [20.0] years) were compared to 145 participants with GCS ≤ 9 (113 male [77.9%]; mean [SD] age: 37.5 [16.3] years) (Table 1). A total of 347 patients with MCTC scores < 3 (255 male [73.5%]; mean [SD] age at baseline: 42.7 [19.2] years) were likewise compared to samples from 70 patients with scores ≥ 3 (56 males [80%]; mean [SD] age at baseline: 19.4) (Table 2). Within the GCS group, the group with GCS ≥ 9 was significantly older (*p* < 0.001), and the GCS < 9 group had a significantly higher injury severity score (ISS) (21.48 [13.524], *p* < 0.001). Within the MCTC group, injury cause was significantly different between groups (*p* < 0.001), and the MCTC ≥ 3 group had a significantly higher injury severity score (ISS) (28.08 [11.900], *p* < 0.001). Age, ISS, and injury cause were controlled for in subsequent analyses.

**Figure 1:**
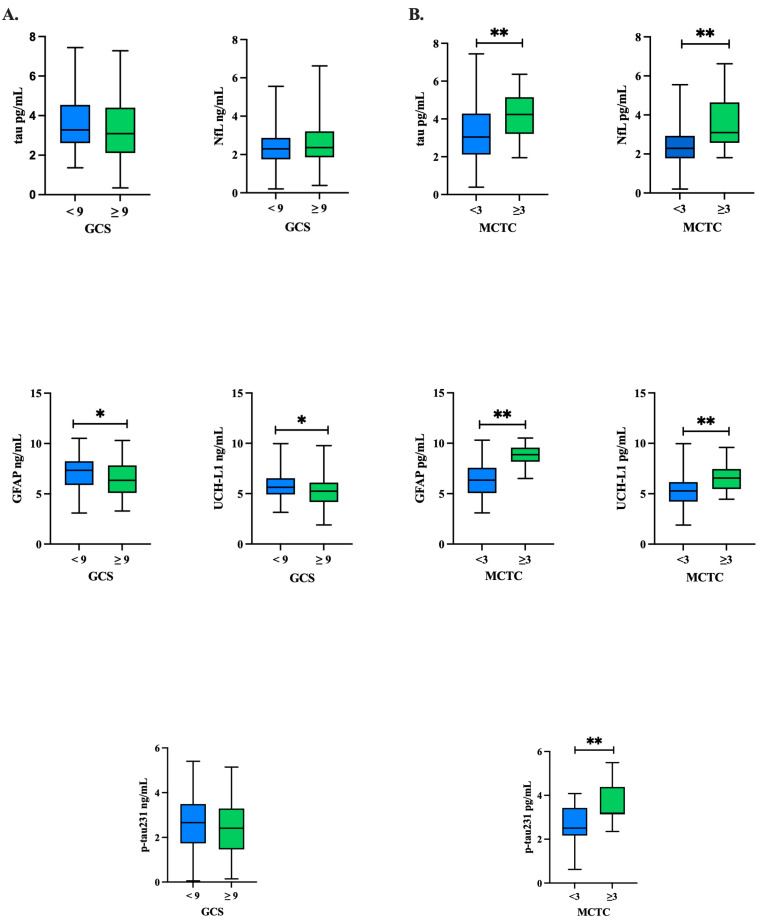
Baseline biomarker concentrations by GCS and MCTC groupings: tau, GFAP, UCH-L1, NfL, p-tau231. Independent *t*-tests were conducted to compare each protein’s concentration between the (**A**) GCS groups and (**B**) MCTC groups at baseline for tau, NfL, GFAP, UCH-L1, and p-tau231. Natural log-transformed GFAP and UCH-L1 concentrations trended higher in the GCS < 9 group at *p* < 0.05, but this trend did not hold after adjustment for covariates. All natural log-transformed protein concentrations trended higher in the MCTC group at *p* < 0.001; NfL, GFAP, and p-tau231 remained significant after adjusting for covariates. GCS: Glasgow Coma Scale; MCTC: Marshall CT classification of traumatic brain injury; GFAP: glial fibrillary acidic protein; NfL: neurofilament light; p-tau231: phosphorylated tau 231; SD: standard deviation; UCH-L1: ubiquitin C-terminal hydrolase L1; * *p* value significant at the *p* < 0.05 level; ** *p* value significant at the *p* < 0.001 level.

Natural log-transformed tau, NfL, GFAP, UCH-L1, and p-tau concentrations were compared between GCS groups (Table 3) and MCTC groups (Table 4) using independent *t*-tests. The comparison of biomarker concentrations in patients with a GCS ≥ 9 compared to GCS < 9 showed that, overall, there were trends of elevated GFAP (*p* = 0.019) and UCH-L1 (*p* = 0.006) in the GCS < 9 group (Figure 1A, Table 3). Yet, these comparisons were not significant when adjusted for covariates (age, ISS, injury cause) in logistic regression analyses (*p*’s > 0.05) (Table 5). In comparing the MCTC groupings of those ≥3 to <3, we found that all biomarker concentrations were significantly higher in the MCTC ≥ 3 group as compared to <3 (*p*’s < 0.001) (Figure 1B, Table 4), and NfL (*p* < 0.001), GFAP (*p* < 0.001), and p-tau231 (*p* < 0.025) remained significant after adjusting for covariates (age, ISS, injury cause) in logistic regression analyses (Table 6).

**Figure 2:**
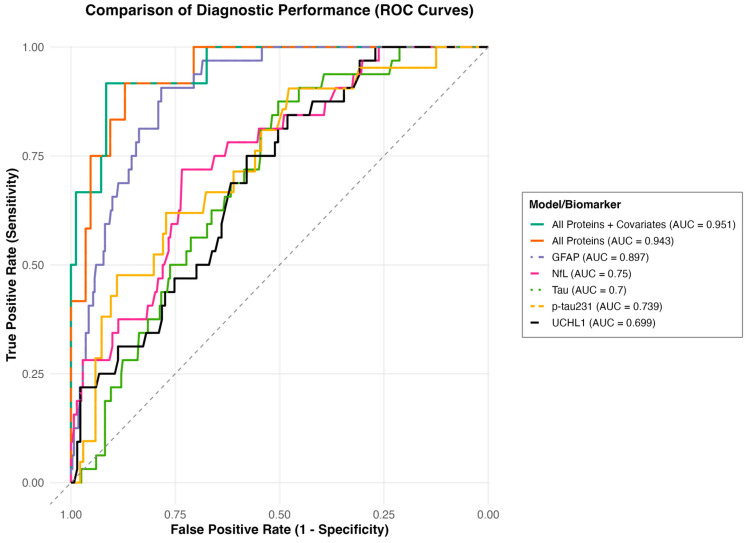
Sensitivity of acute proteins to predict MCTC group. Receiver operating characteristic (ROC) curves stratifying MCTC ≥ 3 vs. <3 for tau, Nfl, GFAP, UCH-L1, and p-tau231 and combined models that include all proteins and all proteins and covariates.

To determine the ability of the proteins to differentiate MCTC groupings, an area under the curve (AUC) analysis was performed (Figure 2). In stratifying MCTC ≥ 3 vs. <3, GFAP, NfL, tau, and p-tau231 significantly predicted MCTC ≥ 3 group and had fair to good AUC values [GFAP (AUC 0.897, 95% CI 0.853–0.940); NfL (AUC 0.75, 95% CI 0.666–0.834); tau (AUC 0.7, 95% CI 0.621–0.780); p-tau231 (AUC 0.739, 95% CI 0.627–0.852)]. The combined proteins model showed good discriminatory ability to predict MCTC ≥ 3 group (AUC 0.943, 95% CI 0.888–0.998), and the combined proteins and covariates model showed good ability to predict the MCTC ≥ 3 group (AUC 0.951, 95% CI 0.894–1.000).

## 3. Discussion

This study reports that GCSs do not relate to biomarker levels acutely following a TBI with a range of severity, whereas the MCTC is highly significant. This finding highlights the value of the recently proposed new framework for the characterization of acute TBI, which instead of using the GCS to group patients, incorporates four pillars: a clinical pillar encompassing the full GCS and pupillary response; a biomarker pillar reflecting blood-based measures of injury; an imaging pillar representing pathoanatomical features; and a modifier pillar accounting for variables that may alter clinical presentation and recovery trajectories (CBI-M framework) [45]. Thus, the findings presented here provide further evidence of the need to better characterize pathologies within TBI, and specifically highlight how Marshall CT scoring or similar methods may provide new directions into the development of clinically relevant biomarker models that reflect underlying structural injury across the spectrum of TBI.

Specifically, the findings that higher plasma concentrations of GFAP, NfL, and p-tau231 measured within one hour of moderate-to-severe TBI were associated with an MCTC score ≥ 3, but not with GCS, suggest that these markers may reflect early structural injury detectable on neuroimaging. Our findings are consistent with prior work examining GFAP, UCH-L1, NfL, and tau in relation to GCS and Marshall CT classification (MCTC) in acute TBI patients [46,47]. In one study, higher serum biomarker levels at admission correlated with lower GCS and higher MCTC scores, although after adjustment, only NfL remained associated with GCS, and both NfL and GFAP with MCTC [46]. In our analysis, no biomarkers were associated with GCS after adjustment, whereas GFAP, NfL, and p-tau231 remained significant for MCTC. Similarly, the multicenter CENTER-TBI study reported that serum GFAP, NfL, and UCH-L1 measured ≤ 24 h after injury improved prognostic performance for moderate-to-severe TBI patients with a Marshall score of <3 and ≥3 [47]. These prior studies did not assess p-tau231 and differences in injury severity, time of sample collection, and classification approach may contribute to the differences in findings. Collectively, these results suggest that blood-based biomarkers and CT reflect overlapping yet distinct aspects of TBI pathology.

Elevated GFAP may relate to astroglial injury accompanying parenchymal contusion, edema, or mass effect that typifies MCTC ≥ 3 lesions. This observation aligns with prior studies demonstrating that GFAP concentrations correlate with the presence and extent of CT-positive lesions and with unfavorable outcomes following TBI [16,48,49,50]. Similarly, early increases in NfL support the interpretation that diffuse axonal injury contributes to severity captured by higher MCTC scores, consistent with findings from prior work showing that plasma NfL predicts lesion burden and long-term degeneration [51]. The association between p-tau231 and MCTC ≥ 3 suggests that microtubule disruption and early tau hyperphosphorylation may occur in proportion to structural damage severity, aligning with recent evidence linking p-tau231 with neuronal cytoskeletal injury and outcome in severe TBI [18]. UCH-L1 and total tau were not significantly associated with MCTC severity in our cohort. UCH-L1 has been reported to rise rapidly but transiently following TBI, with diagnostic sensitivity highest at 2–8 h post-injury [16,49]. Thus, it is possible our sampling time may have preceded its peak. Likewise, total tau may lack the sensitivity of phosphorylated tau species for detecting microtubule injury specific to severe lesions, as non-phosphorylated tau can originate from multiple cell types [18,52,53].

These findings show that developing biomarkers beyond differentiating of GCS groupings or CT as a dichotomous variable may lead to clinical relevance of biomarkers in characterizing the pathological processes related to acute injuries. Inclusion of both the GCS and MCTC provided a more comprehensive characterization of injury severity, recognizing that functional impairment (GCS) and structural damage (MCTC) represent distinct yet interrelated dimensions of TBI pathophysiology. These metrics should not be considered equivalent from a biomarker perspective. Because the biomarkers examined here primarily reflect cellular and structural injury processes, stronger associations with MCTC than with GCS are consistent with prior findings showing that neuroimaging indices of lesion burden more directly capture the underlying tissue disruption measured by these biomarkers [46,47,54]. The Marshall CT scoring provides an index of risk for mortality and morbidity, as well as a categorization of injury type and requirement for neurosurgical intervention; for example, individuals with an MCTC of 5 have previously been reported to have fewer deaths compared to those with a score of 6 [32,33]. To this end, the greater range of information conveyed by the Marshall CT scoring classification compared to a dichotomous CT evaluation allows for more useful relationships to be established between blood-based biomarkers, injury severity based on score, and post-injury outcomes, with potential utility to integrate within clinical care.

Our finding that GFAP is only significant in the Marshall CT scoring method and not GCS across a range of severities is an important finding, as most studies primarily include one severity group, not allowing for an evaluation of biomarker performance across severities. It also supports the assertation that in some patients, there could be quickly evolving pathologies that are not reflective in neurological functioning, and then result in high mortality related to increased blood volume and pressure, and life-critical reductions in physiological functioning. The risk for quick escalation of pathology in the absence of clinical declines has been observed in patients with GCS > 13, but with subdural hematomas that do not alter consciousness until severe [1,55]. These risks become more substantial in deployment settings in which CT imaging requires transport, and when delayed due to a higher GCS score, could lead to mortality risks. Thus, there may be utility for the development of triaging biomarker capabilities for GFAP.

Mortality rates from severe traumatic brain injuries (TBIs) range from one-quarter to one-third of those injured, a rate that has remained stable, while other acute injuries to the brain such as stroke have dramatically reduced by more than 30% in the past few decades [56,57]. In contrast to stroke, acute medical management of TBIs has not benefited from the integration of fluid biomarkers to diagnose TBIs, or to direct acute medical care. For example, in stroke care, blood-based biomarkers including GFAP and NfL are used to assess injury severity and guide treatment decisions. GFAP can help distinguish between ischemic and hemorrhagic stroke, while NfL levels correlate with neuronal damage and long-term functional outcomes [58].

Together, our findings lead us to consider other methods to improve the alignment of fluid and neuroimaging biomarkers within acute care for TBI patients. These findings reinforce the need to move beyond GCS-only frameworks and toward multi-dimensional classification systems, as recently advocated by the CENTER-TBI and TRACK-TBI consortia [45]. Firstly, by demonstrating that Marshall CT classification shows significant associations with blood-based biomarkers, the current findings support using more granular CT-based scoring (rather than simple CT+ vs. CT−) to refine prognostic models. Previous work has shown that MCTC scores correlate with mortality and functional outcomes [32,33]. Combining this with biomarker information could improve the prediction of outcomes such as intracranial hypertension, the need for surgery, and long-term disability. Secondly, previous studies have noted variability in GFAP/UCH-L1 levels in mild TBI, where CT findings may be negative but microscopic injuries still occur [4]. This highlights the need for refined reference standards for biomarker studies, potentially using more granular imaging classifications instead of binary CT outcomes. Thirdly, there is the potential to guide therapeutic decisions and triage. For example, in stroke, biomarker-informed severity indexing has contributed to faster interventions and improved mortality rates [58,59]. Similar approaches in TBI could enable improved triage decisions, such as identifying patients who may benefit from early decompressive craniectomy or intensive monitoring. The nuanced relationship between MCTC scores and biomarker levels may inform real-time risk stratification tools in emergency and critical care. Finally, these results underscore the need for additional prospective studies that align blood biomarkers with detailed imaging phenotypes and clinical trajectories. Large-scale efforts like CENTER-TBI and TRACK-TBI already collect this type of integrated data, and the current findings could contribute to refining analytic frameworks to generate clinically actionable biomarker thresholds.

There are several limitations to the current study. Firstly, while the analysis demonstrates that MCTC scoring shows stronger associations with blood-based biomarkers than GCS groupings, the generalizability of these findings may be limited by sample size and the potential for selection bias; for example, some participants requiring neurosurgery were not available for baseline blood draws. Secondly, although the study highlights the relationship between CT-based injury classification and biomarker levels, the temporal dynamics of biomarker expression were not examined for this study and may influence interpretation. Further examination of biomarker concentrations over time is planned for future analyses. Thirdly, the lack of long-term outcome data in the current analyses limits conclusions about the prognostic utility of the biomarker–CT relationships. Fourthly, although this study dichotomizes the MCTC as supported in prior work, there is currently no clear consensus among published studies regarding the optimal method for analyzing the MCTC together with fluid biomarkers for outcome prediction. Although outside the scope of the current study, future work could explore other methods including ordinal sensitivity analyses. Finally, the study focuses on five biomarkers: tau, NfL, GFAP, UCH-L1, and p-tau231, and does not account for other potential markers that may exhibit patterns of association with CT findings or clinical severity.

## 4. Materials and Methods

### 4.1. Clinical Methods

This study includes a subset of subjects from the phase II double-blind, multicenter randomized controlled trial, “Prehospital Tranexamic Acid Use for Traumatic Brain Injury” (TXA for TBI; ClinicalTrials.gov NCT01990768) [60]. We limited our investigation to the subjects in the placebo arm of the parent trial. A total of 966 participants were enrolled in the parent study. A total of 417 participants were in the placebo arm with blood samples drawn within 1 h of injury and biomarker data available for analysis. Inclusion criteria for the TXA parent trial were adult patients with a TBI defined as a GCS score of 3–12 who were not in shock (systolic blood pressure ≥ 90 mm Hg). Patients were excluded if they had a prehospital GCS score of 3 with no reactive pupils; an estimated injury-to-treatment time greater than 2 h or an unknown time of injury; clinical suspicion of seizure activity, acute myocardial infarction, or stroke; a known history of seizures, thromboembolic disorders, or renal dialysis; received CPR by EMS before randomization; sustained burns covering more than 20% total body surface area; or were pregnant or incarcerated (suspected or known). Subjects were recruited by 20 trauma centers within the United States or Canada between May 2015 and March 2017. Blood samples were obtained (upon arrival to the ED [0 h]) and centrifuged, aliquoted, frozen at −80 °C, bar coded, and batch shipped by the clinical research staff to the Trauma Research Institute of Oregon, Oregon Health and Science University (OHSU). As part of the placebo arm of the parent trial, all subjects received a bolus dose of 250 mL of 0.9% sodium chloride administered in the prehospital setting, followed by a second 250 mL infusion administered over 8 h after arrival to hospital.

A head CT was performed upon arrival. Digital images of head CTs were obtained in Digital Imaging and Communication in Medicine format and transferred to the OHSU image repository in a de-identified manner. Head CT scans were reviewed centrally at OHSU by a neuroradiologist-trained technician using software designed to obtain computerized measurements.

Neurological and injury severity were characterized using standardized clinical and radiological measures. The Glasgow Coma Scale (GCS) was used to assess level of consciousness on admission, based on eye-opening, verbal, and motor responses, yielding a total score ranging from 3 to 15, with lower scores indicating greater impairment [21]. The Marshall CT classification (MCTC) was applied to the admission head CT scan to grade structural injury severity according to basal cistern status, degree of midline shift, and presence or absence of high- or mixed-density mass lesions > 25 cc, resulting in six categories from Diffuse Injury I (no visible pathology) to VI (non-evacuated mass lesion) (Table A1) [30]. Participants were stratified into groups based on GCS ≥ 9 (*n* = 271) and <9 (*n* = 145), and MCTC < 3 (*n* = 347) and ≥3 (*n* = 70). The GCS and the MCTC were included in analyses to capture complementary domains of TBI, with the GCS reflecting functional neurological status and the MCTC representing structural injury on neuroimaging. The injury severity score (ISS) was calculated as an anatomical composite derived from the Abbreviated Injury Scale (AIS), using the sum of squares of the highest AIS scores from the three most severely injured body regions [61]. The ISS ranges from 1 to 75, with higher scores reflecting greater overall injury burden and scores > 15 conventionally defining major trauma.

### 4.2. Biomarker Methods

Simoa technology, developed by Quanterix^®^, Inc., incorporates an array of femtoliter-sized reaction chambers that can isolate and detect single enzyme molecules, measuring low-abundance proteins, which cannot be detected with a regular ELISA system. The Simoa HD-X Analyzer enables protein concentrations by isolating and detecting single immunocomplexes in arrays of femtoliter-volume wells, so that proteins are measured at sub-femtomolar concentrations. Quanterix^®^, Inc. (Billerica, MA, USA) developed an ultra-sensitive single-molecule enzyme-linked immunoassay commercially available Neurology 4-Plex A Advantage kit that measures total tau, neurofilament light (NfL), glial fibrillary acidic protein (GFAP), and ubiquitin carboxyl-terminal hydrolase L1 (UCH-L1) simultaneously for low-abundance levels of all four proteins (Simoa™, Quanterix Corporation, Lexington, MA, USA). Samples were run in duplicate as well as calibrators and controls with an in-instrument 4× dilution. Results are produced by the digital immunoassay as well as calibration curve reports. The lower limit of detections for tau, Nf-L, GFAP, and UCH-L1 were 0.024 pg/mL, 0.104 pg/mL, 0.221 pg/mL, and 1.74 pg/mL, respectively.

### 4.3. Statistical Methods

All GCS and MCTC group comparisons were undertaken including all sample data, including outliers. To ensure that outliers were not the primary driver in study findings, we repeated analyses excluding values greater than 2 standard deviations from the mean. Further, due to the high variation in the data, the figures generated include values that were natural log-transformed to appropriately show group differences while also reflecting the distribution of the data. Analysis of variance (ANOVA) and Chi-square tests were performed to determine group differences in demographic characteristics. Log-transformed protein concentrations were compared between groups using independent *t*-tests. Binomial logistic regression analyses were subsequently run, controlling for significant covariates as determined by group demographic analyses, age, injury severity score, and injury cause, to determine group prediction. AUC and the optimal cutoff values were calculated using the pROC (v1.18.5) package in R (v4.4.0). Generalized Linear Models (GLMs) were employed to identify optimal combinations of biomarkers for classification tasks. The GLM was trained using protein expression data as predictors and the corresponding grouping data as the response variable, enabling the model to establish relationships between biomarker expression and group classifications. After training, the predictions generated by the GLM underwent an additional area under the receiver operating characteristic curve (AUC) analysis to determine optimal cutoffs. These cutoffs were used to assess key performance metrics, Sensitivity, Specificity, Precision, and Negative Predictive Value (NPV). General linear models adjusted for covariates provided the individual and combined biomarker model AUC results. Analyses were conducted using SPSS V29.0 (Armonk, NY, USA: IBM Corp.) and GraphPad Prism 10.5.0 (La Jolla, CA, USA: GraphPad Software).

## 5. Conclusions

In conclusion, the present findings underscore the clinical utility of the Marshall CT classification system in capturing neuronal injury features that align with blood-based biomarker changes, providing a more nuanced alternative to traditional GCS or dichotomous CT groupings. These results support the growing emphasis on integrated, multi-domain classification frameworks for acute TBI that combine clinical, imaging, and biomarker data. Future studies are needed to explore how such integrated models may improve diagnostic precision, prognostic accuracy, and treatment decision-making in patients with acute TBI.

## Figures and Tables

**Table 1:** Demographics and clinical GCS grouping.

	GCS ≥ 9	GCS < 9	*p*
Age in years, M (SD)	44.4 (20.0)	37.5 (16.3)	<0.001 ^a^**
Sex, No. (%)			0.430 ^b^
Male	207 (74.5)	113 (77.9)	
Female	71 (25.5)	32 (22.1)	
Race, No. (%)			0.265 ^b^
American Indian/Alaskan Native/Aboriginals	4 (1.4)	0 (0)	
Asian	7 (2.5)	4 (2.8)	
Black/African American	44 (15.8)	22 (15.2)	
Native Hawaiian/Other Pacific Islander	0 (0)	1 (0.7)	
White	200 (71.9)	100 (69.0)	
More than one race	2 (0.7)	0 (0)	
Unknown status	21 (7.6)	18 (12.4)	
Ethnicity, No. (%)			0.403 ^b^
Non-Hispanic	211 (75.9)	110 (75.9)	
Hispanic	40 (14.4)	16 (11.)	
Unknown status	27 (9.7)	19 (13.1)	
Injury cause, No. (%)			0.133 ^b^
MVC occupant	87 (31.5)	66 (45.5)	
MVC pedestrian	44 (15.9)	19 (13.1)	
MVC bicycle	17 (6.2)	7 (4.8)	
MVC motorcycle or off-road	27 (9.8)	9 (6.2)	
Suicide	5 (1.8)	5 (3.4)	
Assault	23 (8.3)	10 (6.9)	
Ground-level fall	42 (15.2)	13 (9.0)	
Fall from height (>1 m)	26 (9.4)	15 (10.3)	
Other cause	5 (1.8)	1 (0.7)	
Injury severity score, M (SD)	15.25 (11.189)	21.48 (13.524)	<0.001 ^a^**
GCS, M (SD)	10.94 (0.985)	5.36 (1.766)	<0.001 ^a^**

Note. The percentages in each column refer to the proportion of individuals in each sex, race, ethnicity, and injury cause category. GCS: Glasgow Coma Scale; M: mean; MVC: motor vehicle collision; SD: standard deviation; ^a^ ANOVA; ^b^ Pearson’s chi square; ** *p* value significant at the *p* < 0.001 level.

**Table 2:** Demographics and clinical MCTC grouping.

	MCTC < 3	MCTC ≥ 3	*p*
Age in years, M (SD)	42.7 (19.2)	42.0 (19.4)	0.792 ^a^
Sex, No. (%)			0.254 ^b^
Male	255 (73.5)	56 (80.0)	
Female	92 (26.5)	14 (20.0)	
Race, No. (%)			0.337 ^b^
American Indian/Alaskan Native/Aboriginals	4 (1.2)	0 (0)	
Asian	8 (2.3)	2 (2.9)	
Black/African American	56 (16.1)	9 (12.9)	
Native Hawaiian/Other Pacific Islander	0 (0)	1 (1.4)	
White	244 (70.3)	52 (74.3)	
More than one race	2 (0.6)	0 (0)	
Unknown status	33 (9.5)	6 (8.6)	
Ethnicity, No. (%)			0.864 ^b^
Non-Hispanic	263 (75.8)	52 (74.3)	
Hispanic	47 (13.5)	9 (12.9)	
Unknown status	37 (10.7)	9 (12.9)	
Injury cause, No. (%)			<0.001 ^b^**
MVC occupant	134 (38.8)	16 (22.9)	
MVC pedestrian	53 (15.4)	9 (12.9)	
MVC bicycle	18 (5.2)	4 (5.7)	
MVC motorcycle or off-road	29 (8.4)	7 (10.0)	
Suicide	3 (0.9)	7 (10.0)	
Assault	24 (7.0)	7 (10.0)	
Ground-level fall	50 (14.5)	7 (10.0)	
Fall from height (>1 m)	33 (9.6)	9 (12.9)	
Other cause	1 (0.3)	4 (5.7)	
Injury severity score, M (SD)	15.33 (11.878)	28.08 (11.900)	<0.001 ^a^**
GCS, M (SD)	9.47 (2.663)	6.84 (3.355)	<0.001 ^a^**

Note. The percentages in each column refer to the proportion of individuals in each sex, race, ethnicity, and injury cause category. GCS: Glasgow Coma Scale; M: mean; MVC: motor vehicle collision; SD: standard deviation; ^a^ ANOVA; ^b^ Pearson’s chi square; ** *p* value significant at the *p* < 0.001 level.

**Table 3:** Natural log-transformed biomarker concentrations in the GCS groups.

Biomarker, M (SD)	GCS ≥ 9	GCS < 9	*p*
Tau	3.31 (1.52)	3.59 (1.36)	0.140
NfL	2.58 (1.07)	2.40 (0.95)	0.180
GFAP	6.49 (1.72)	7.01 (1.77)	0.019 *
UCH-L1	5.47 (1.16)	5.94 (1.02)	0.006 *
p-tau231	2.50 (1.13)	2.66 (1.15)	0.382

Note. GCS: Glasgow Coma Scale; GFAP: glial fibrillary acidic protein; NfL: neurofilament light; p-tau231: phosphorylated tau 231; SD: standard deviation; UCH-L1: ubiquitin C-terminal hydrolase L1; * *p* value significant at the *p* < 0.05 level.

**Table 4:** Natural log-transformed biomarker concentrations in the MCTC groups.

Biomarker, M (SD)	MCTC < 3	MCTC ≥ 3	*p*
Tau	3.27 (1.47)	4.19 (1.16)	<0.001 **
NfL	2.44 (0.96)	3.55 (1.36)	<0.001 **
GFAP	6.37 (1.63)	8.84 (0.94)	<0.001 **
UCH-L1	5.61 (1.10)	6.40 (0.84)	<0.001 **
p-tau231	2.41 (1.12)	3.34 (0.96)	<0.001 **

Note. MCTC: Marshall CT classification of traumatic brain injury; GFAP: glial fibrillary acidic protein; NfL: neurofilament light; p-tau231: phosphorylated tau 231; SD: standard deviation; UCH-L1: ubiquitin C-terminal hydrolase L1; ** *p* value significant at the *p* < 0.001 level.

**Table 5:** Logistic regression analysis of GCS groupings.

Biomarker	Independent Variable	B (SE)	Wald X^2^	OR (95% CI)	*p*
Tau	Intercept	1.028 (0.128)	64.588	2.795	<0.001
	Tau	0.094 (0.124)	0.571	1.098	0.450
	Age (years)	0.019 (0.009)	4.653	1.019	0.031
	Injury Severity Score	−0.028 (0.014)	4.003	0.972	0.045
	Injury Cause		4.638		0.865
	*n* = 315				
NfL	Intercept	1.028 (0.128)	64.588	2.795	<0.001
	NfL	0.161 (0.175)	0.846	1.174	0.358
	Age (years)	0.013 (0.009)	2.120	1.014	0.145
	Injury Severity Score	−0.028 (0.013)	4.522	0.972	0.033
	Injury Cause		4.459		0.879
	*n* = 315				
GFAP	Intercept	1.028 (0.128)	64.588	2.795	<0.001
	GFAP	−1.31 (0.094)	1.925	0.877	0.165
	Age (years)	0.017 (0.008)	3.999	1.017	0.046
	Injury Severity Score	−0.011 (0.014)	0.648	0.989	0.421
	Injury Cause		4.547		0.872
	*n* = 315				
UCH-L1	Intercept	−0.355 (0.158)	5.044	0.701	0.025
	UCH-L1	−0.278 (0.191)	2.121	0.758	0.145
	Age (years)	0.004 (0.011)	0.132	1.004	0.716
	Injury Severity Score	−0.028 (0.019)	2.135	0.972	0.144
	Injury Cause		5.268		0.810
	*n* = 165				
p-tau231	Intercept	−0.220 (0.162)	1.857	0.802	0.173
	p-tau231	0.115 (0.200)	0.327	1.121	0.568
	Age (years)	0.007 (0.012)	0.327	1.007	0.545
	Injury Severity Score	−0.028 (0.018)	2.306	0.973	0.129
	Injury Cause		5.533		0.786
	*n* = 155				

Note. B = unstandardized regression coefficient; SE = standard error; OR = odds ratio; CI = confidence interval.

**Table 6:** Logistic regression analysis of MCTC groupings.

Biomarker	Independent Variable	B (SE)	Wald X^2^	OR (95% CI)	*p*
Tau	Intercept	−2.194 (0.189)	134.207	0.112	<0.001
	Tau	0.187 (0.197)	0.899	1.206	0.343
	Age (years)	−0.013 (0.014)	0.907	0.987	0.341
	Injury Severity Score	0.089 (0.021)	18.314	1.094	<0.001
	Injury Cause		14.712		0.099
	*n* = 309				
NfL	Intercept	−2.194 (0.189)	134.207	0.112	<0.001
	NfL	0.978 (0.247)	15.704	2.659	<0.001
	Age (years)	−0.036 (0.015)	5.724	0.965	0.017
	Injury Severity Score	0.079 (0.020)	15.274	1.082	<0.001
	Injury Cause		9.952		0.354
	*n* = 309				
GFAP	Intercept	−2.194 (0.189)	134.207	0.112	<0.001
	GFAP	1.128 (0.226)	25.009	3.090	<0.001
	Age (years)	−0.009 (0.014)	0.387	0.991	0.534
	Injury Severity Score	0.053 (0.022)	6.000	1.055	0.014
	Injury Cause		10.519		0.310
	*n* = 309				
UCH-L1	Intercept	−1.409 (0.197)	51.090	0.244	<0.001
	UCH-L1	0.541 (0.278)	3.790	1.718	0.052
	Age (years)	−0.019 (0.014)	1.652	0.982	0.199
	Injury Severity Score	0.066 (0.022)	8.813	1.068	0.003
	Injury Cause		14.395		0.109
	*n* = 163				
p-tau231	Intercept	−1.853 (0.235)	62.358	0.157	<0.001
	p-tau231	0.761 (0.340)	5.000	2.140	0.025
	Age (years)	0.007 (0.017)	0.174	1.007	0.676
	Injury Severity Score	0.069 (0.024)	7.943	1.071	0.005
	Injury Cause		6.703		0.569
	*n* = 155				

Note. B = unstandardized regression coefficient; SE = standard error; OR = odds ratio; CI = confidence interval.

## Data Availability

The data presented in this study are available on request from the corresponding author.

## Appendix A

**Table A1:** Marshall scoring system.

Score	Category	Definition
1	Diffuse Injury I (no visible pathologic change)	No visible intracranial pathology seen on CT scan
2	Diffuse Injury II	Cisterns present with midline shift of 0–5 mm and/or lesions densities present; no high- or mixed-density lesion > 25 mL; may include bone fragments and foreign bodies
3	Diffuse Injury III (swelling)	Cisterns compressed or absent with midline shift 0–5 mm; no high- or mixed-density lesion > 25 mL
4	Diffuse Injury IV (shift)	Midline shift > 5 mm; no high- or mixed-density lesion > 25 mL
5	Evacuated Mass Lesion	Any surgically evacuated lesion
6	Non-evacuated Mass Lesion	High- or mixed-density lesion > 25 mL; not surgically evacuated

Note. Adapted from Marshall et al., 1992 [30].
